# The Effect of a 10-MDP-Based Dentin Adhesive as Alternative for Bonding to Implant Abutment Materials

**DOI:** 10.3390/ma15155449

**Published:** 2022-08-08

**Authors:** Paula C. K. Carvalho, Cláudia C M S Almeida, Rodrigo O. A. Souza, Rubens Nisie Tango

**Affiliations:** 1 Department of Dental Materials and Prosthodontics, Institute of Science and Technology of São José dos Campos, São Paulo State University, São José dos Campos 12245-000, Brazil; 2 Private Dental Office, Recife 51111-010, Brazil; 3 Dentistry Department, Federal University of Rio Grande do Norte, Natal 59056-000, Brazil

**Keywords:** 10-MDP, bond strength, resin cement, titanium, dentistry

## Abstract

Bonding to different dental restorative materials is challenging. This study aimed to evaluate the effect of a 10-MDP-based dentin adhesive on the shear bond strength (SBS) of self-adhesive resin cement (RC) to implant abutment materials. One hundred and twenty specimens were obtained from zirconia (ZO), cobalt-chromium alloy (CoCr), and commercially pure titanium (Ti), which were treated as follows (n = 10): control group—non-treated (CG), 10-MDP-based dentin adhesive (SB), light-cured SB (SB-LC), and zirconia primer (ZP). Blocks of RC were buildup and, after 24 h, were tested for bond strength. Data of SBS (MPa) were submitted to two-way ANOVA and Tukey test (α = 0.05). There was no difference in SBS among materials for CG and ZP, higher SBS were recorded for Ti SB and Ti SB-LC compared to ZO upon the same surface treatments. For the comparisons among treatments, SB-LC showed the highest SBS for CoCr. For ZO and Ti, higher SBS were recorded with SB and SB-LC. No cohesive failures were observed. It was concluded that the surface treatment with 10-MDP-based materials increased the bond strength of the resin cement to abutment materials, which showed to be material dependent.

## 1. Introduction

Implant-supported oral rehabilitation provides advantages in the stability and retention of both single and multi-unit prostheses compared to removable prostheses [1]. Implant-supported fixed prostheses can be screw-retained or cemented to an intermediary abutment, which can be made of different materials such as titanium (Ti), cobalt-chromium (CoCr), and zirconia (ZO), among others [2,3].

Cemented prostheses are commonly indicated for short-space prostheses with margins at or above the mucosa level, in cases with a narrow diameter crown, and for patients with limited jaw opening [2]. Additionally, it can also be considered with a deeper prosthetic finish line that allows the removal of excess cement. However, it has been demonstrated that extraoral cementation significantly decreases the amount of cement excess [4]. Many surface treatments, such as sandblasting, silicatization, plasma spraying, primer application, and laser etching [5,6,7,8,9,10,11,12], have been suggested to achieve higher bond strength between resin cement and dental ceramics for dental-supported restorations. Sandblasting metal oxide-based substrates with aluminum oxide particles (Al_2_O_3_) or silica-coated particles demands specific equipment. Thus, chemical treatments are more likely to be applied in clinical routines.

Products containing the monomer 10-methacryloyloxydecyl dihydrogen phosphate (10-MDP) have been indicated for the surface treatment of ZO; hence it presents a high affinity with metal oxides [13,14]. The 10-MDP is a phosphate functional monomer commonly used in primers, universal dentin adhesives, and resin cement to optimize the bond strength by chemical adhesion by establishing covalent bonds between ceramics and the resin cement [14,15,16].

Resin cement is the most used cementation agent because of the high bond strength they promote [16], and more recently, self-adhesive resin cement containing 10-MDP or other phosphate functional monomers have been introduced [14,16,17]. Self-adhesive resin cements are easy to handle [18], and with higher bond strength to zirconia, metallic alloys are obtained in comparison to conventional resin cements [19].

Taking into account the afore-mentioned chemical bonding of 10-MDP to metal oxides, its use could be considered for the surface treatment of Ti and CoCr. However, there are poor information about this use in the literature [14,15]. Thus, this study aimed to evaluate the effect of a 10-MDP-based dentin adhesive on the shear bond strength (SBS) of self-adhesive resin cement to implant abutment materials. The null hypotheses were that: there were no differences in the bond strength among materials, and there were no differences among different surface treatments.

## 2. Materials and Methods

One hundred and twenty specimens of c.p. titanium (Ti), cobalt-chromium alloy (CoCr), and zirconia (ZO) blocks (n = 10) were obtained for the study (Table A1 in Appendix A). Ti and CoCr specimens were 5 mm in diameter and 7 mm thick, and 7 mm in diameter and thickness, respectively. ZO specimens (7 mm thick) were cut from CAD blocks using a double-sided diamond disc (No. 34570, Microdont, Sao Paulo, Brazil) mounted on a handpiece (LB100 Beltec, Sao Paulo, Brazil). Each specimen was finished sequentially in a grinding machine (Labpol 8–12, Extec, Enfield, CT, USA) with sandpapers (grit # 600, # 800, and # 1200) under water cooling. Prior to sintering (Furnace 200, 3M ESPE, Seefeld, Germany), according to the manufacturer’s recommendations, specimens were sonicated (Cristófoli Biosafety Equipment, Paraná, Brazil) in distilled water for 5 min.

Specimens of each material (Ti, CoCr, and ZO) were embedded in self-cured acrylic resin (JET, Classic Dental, São Paulo, Brazil) using an industrial silicone mold (Silicone Master, Talmax, Curitiba, Parana, Brazil). Specimens were divided into subgroups according to surface treatment as follows: Control group (CG): with no treatment; 10-MDP-based dentin adhesive Single Bond Universal (SB): a layer of adhesive was actively applied for 10 s with a disposable brush and blow-dried for 5 s with no light curing; Single Bond Universal light-cured (SB-LC): a layer of adhesive was applied as above and was light-cured for 20 s (1200 mW/cm^2^, Radii Cal, SDI, Victoria, Australia); and Z-prime (ZP): a layer of Z-prime was applied for 10 s and blow-dried for 5 s.

Soon after the surface treatment, blocks of self-adhesive resin cement (RelyX U200) were buildup using a Teflon mold (2 mm high, 2.37 mm diameter, Ultradent, South Jordan, UT, USA) held in position with a metal clip (Ultradent), and were light-cured for 20 s. Specimens were stored in distilled water for 24 h at 37 °C prior to the SBS test, which was performed in a universal testing machine (ODEME, Ribeirao Preto, SP, Brazil) using a blade-shaped metal device at a crosshead speed of 0.5 mm/min until fracture [8]. Pre-test failed specimens were substituted with new specimens.

The SBS was calculated by the formula: R = F/A, where R = bond strength (MPa); F = force (N); A = interfacial area (mm). The adhesive area was defined by the area of a circle, calculated by the formula: A = πr^2^, where π = 3.14 and r (radius) = 1.185 mm. Using this formula, the cross-sectional area was 4.40 mm². SBS (MPa) data were analyzed using two-way ANOVA and Tukey’s test, both with α = 0.05 (Statistix version 8.0, Analytical Software, Tallahassee, FL, USA).

The fractured specimens were analyzed in a stereomicroscope under 20× magnification (Stereo Discovery V20, Zeiss, Göttingen, Germany) and the failure pattern was classified as adhesive (A)—failure at the cement/material interface, with no cement residue on the material; cohesive (C)—failure in cement, and mixed (M)—failure at the cement/material interface, with cement residue on the material.

## 3. Results

The results of ANOVA showed the significance of factors and interaction (*p* = 0.000) (Table A2). Mean SBS (MPa) values, standard deviation, and comparisons among groups are presented in Table A3. There was no difference in SBS among materials for CG and ZP, while higher SBS were recorded for Ti SB and Ti SB-LC compared to ZO upon the same surface treatments. CoCr treated with SB presented similar SBS compared to ZO and presented intermediate and similar SBS compared to other materials when treated with SB-LC. For the comparisons among surface treatments, SB-LC showed the highest SBS for CoCr, while the other treatments were similar to each other. For ZO and Ti, higher SBS were recorded with SB and SB-LC. For ZO, ZP showed intermediate SBS, similar to CG, SB, and SB-LC. For Ti, CG, and ZP presented the lowest SBS, similar to each other.

The failure analysis (Table A4) showed that all the failures were A or M, with no cohesive failures in cement or abutment material. Most failures upon surface treatment were classified as M, except for ZO CG, CoCr CG, and CoCr ZP.

## 4. Discussion

Bonding in dentistry has shown to be challenging. Micromechanical retention and chemical adhesion have been used, aiming to increase the bond strength between different materials [5,20]. In the present study, the application of 10-MDP containing primer and universal dentin adhesive showed to increase the SBS between self-adhesive resin cement and tested materials, corroborating with previous studies [21,22,23,24].

The comparisons among groups (Table A3) showed that there were no differences among ZO, CoCr, and Ti for CG and ZP, while higher SBS values were observed with Ti upon treatment with SB and SB-LC. Thus, the first null hypothesis that there were no differences in the bond strength among materials was partially rejected.

Low values of SBS, ranging from 3.4 to 6.4 MPa, were observed for CG groups, in which the self-adhesive resin cement containing 10-MDP was applied directly, confirming that bonding to ZO, CoCr, and Ti are challenging. Although there were no significant differences among materials, higher SBS, ranging from 7.1 to 10.6 MPa, were observed with ZP, in agreement with previously published data. It has been shown that higher bond strength of resin cements are obtained to Ti surface treated with 10-MDP containing primers or cements when compared to ZO [8,9,25]. It could be suggested that the high viscosity of the resin cement and low wettability of ZP on abutment materials hindered higher SBS values. Additionally, it should be considered that Z-prime presents no inorganic filler particles in its composition (Table A1), which could improve the material toughness.

The treatment of the abutment surface with 10-MDP containing primer (Z primer) and universal dentin adhesive (SB and SB-LC) increased the SBS. Therefore, the second null hypothesis that there were no differences among different surface treatments was rejected. These differences in the effectiveness of surface treatments depending on the material could be attributed to differences in hydrophilicity and wettability among materials [26]. Although studies have shown that the use of 10-MDP containing primers, dentin adhesives, and resin cements are effective with zirconia and metal oxides-based materials [21,27,28,29], the use of RelyX U200 and Z-Prime Plus resulted in lower SBS values compared to SB and SB-LC, in contrast with other studies [30,31]. These results could be due to the lower viscosity of the dentin adhesive, which can better interact with the abutment materials leading to micromechanical retention and chemical bonding.

As expected, because of the high elastic moduli of the abutment materials, the failure analysis (Table A4) showed no cohesive failures. Thus, the SBS test [18,32] was shown to be reliable and a better option compared to the microtensile test [33,34], considering the need to slice the specimens in the latter [22]. Pre-test failures were observed in CG groups (CoCr = 2; Ti = 1; ZO = 3), in Ti SBU = 2, and in Ti SB-LC = 1. The effectiveness of SB and SB-LC could be observed by most failures classified as mixed, i.e., part of adhesive/cement remained on the abutment surface, which means that there was no complete debonding (adhesive failure) from the abutment material.

The results of the present study suggest that using a primer or a universal dentin adhesive containing 10-MDP monomer on implant abutments prior to cementation could be used to improve the bond strength. Some limitations of the present study were that complex abutment designs and mechanical cycling were not tested. Many in vitro studies testing techniques to reduce the excesses of cement of implant-supported prosthesis have been performed, but very few have been tested and validated clinically [3]. Thus, additional studies should be performed aiming to predict the long-term performance in clinical situations.

## 5. Conclusions

Limited by the results of this in vitro study, the following conclusions were drawn:

– The surface treatment of implant abutments with 10-MDP containing universal dentin adhesive increased the shear bond strength of a self-adhesive resin cement;
– The effectiveness of surface treatment of implant abutments with 10-MDP containing primer and universal dentin adhesive was material-dependent.

## Data Availability

Not applicable.

## Appendix A

**Table A1:** List of materials, composition, manufacturers, and batch number.

Material	Composition	Manufacturer	Batch Number
Cobalt-ChromiumAlloy	High fusion alloy consisting of 53 to 67% cobalt, 25 to 32% chromium, 2 to 6% molybdenum, tungsten, silicon, iron, carbon, and manganese	DeguDent (Hanau, Germany)	502967D
Lava Plus High Translucent Zirconia	Partially stabilized tetragonal polycrystalline zirconia with 3mol% yttria, Alumina content less than 0.1%.	3M (Saint Paul, MN, US)	645105
One-step Primer Z-Prime Plus	Ethanol; Bisphenol A diglycidyl ether dimethacrylate (BisGMA); 2-Hydroxyethyl methacrylate; 10-methacryloyloxydecyl dihydrogen phosphate; Triethylamine	Bisco (Schaumburg, IL, US)	1800005511
Self-adhesive resin cement RelyX U200	Glass powder (65997-17-3), surface modified with 2-propenoic, 2-methyl-3- (trimethoxysilyl) propyl (2530-85-0), bulk material; Substituted dimethacrylate; Sodium P-toluenesulfonate; 1,12-Dodecane dimethacrylate; Silane treated silica; 2,4,6 (1H,3H,5H) Pyrimidinetrione, 5-phenyl-1- (phenylmethyl) calcium salt (2:1); Calcium hydroxide; 2-propanoic acid, 2-methyl [(3-methoxypropyl) imino] di-2,1-ethanediyl ester; Methacrylated amine; Titanium dioxide.	3M	4588178
Single Bond Universal	2-hydroxyethyl methacrylate; Bisphenol A diglycidyl ether dimethacrylate (BisGMA); Decamethylene dimethacrylate; Ethanol; Silane treated silica; Water; 1,10-Decanediol phosphate methacrylate; Acrylic Copolymer and Itaconic Acid; Camphorquinone; N, N-Dimethylbenzocaine; 10-methacryloyloxydecyl dihydrogen phosphate	3M ESPE	668204
Titanium Universal Trunnion Analog	Commercially pure titanium (4.5 mm × 6 mm)	Neodent (São Paulo, Brazil)	800360172

**Table A2:** Results of Two-way ANOVA for shear bond strength (MPa).

Effect	df	SS	MS	F	P
Treatment	3	2199.88	733.29	39.54	0.0000
Material	2	701.91	350.95	18.92	0.0000
Treatment × Material	6	513.87	85.646	4.62	0.0003
Residual	108	2002.97	18.546		
Total	119	5418.63			

df: degree of freedom; SS: sum of squares; MS: mean square.

**Table A3:** Mean shear bond strength (MPa) and standard deviations for comparisons among groups.

Material	CG	ZP	SB	SB-LC
CoCr	4.9 (3.5) B, a	7.1 (3.3) B, a	8.7 (6.1) B, b	17.2 (6.4) A, ab
Ti	6.4 (2.9) B, a	10.6 (2.9) B, a	19.7 (4.4) A, a	20.3 (3.8) A, a
ZO	3.4 (2.8) B, a	9.2 (3.7) AB, a	11.9 (5.9) A, b	10.8 (3.3) A, b

Different capital letters in the row and small case letters in the column indicate significant differences between the groups, according to Tukey test (*p* < 0.05). CG = control group, ZP = zirconia primer, SB = Single Bond Universal non-light-cured, SB-LC = Single Bond Universal light-cured, CoCr = cobalt-chromium alloy, Ti = titanium alloy, ZO = zirconia.

**Table A4:** Failure mode (%) of specimens upon shear bond strength test.

GROUPS		Failure Mode (%)			Total (%)
		A	M	C	
CoCr	CG	50	50	0	100
	ZP	50	50	0	100
	SB	0	100	0	100
	SB-LC	0	100	0	100
Ti	CG	20	80	0	100
	ZP	10	90	0	100
	SB	0	100	0	100
	SB-LC	0	100	0	100
ZO	CG	70	30	0	100
	ZP	20	80	0	100
	SB	30	70	0	100
	SB-LC	10	90	0	100

A = Adhesive in cement/material interface; C = Cohesive in cement or material; M = Mixed (adhesive in material/cohesive in cement). CG = control group, ZP = zirconia primer, SB = Single Bond Universal non-light-cured, SB-LC = Single Bond Universal light-cured, CoCr = cobalt-chromium alloy, Ti = titanium alloy, ZO = zirconia.
